# Quantitative PPARγ expression affects the balance between tolerance and immunity

**DOI:** 10.1038/srep26646

**Published:** 2016-05-25

**Authors:** Ya-Hui Liu, Yau-Sheng Tsai, Shih-Chieh Lin, Nan-Shih Liao, Ming-Shiou Jan, Chung-Tiang Liang, Shih-Wen Hsu, Wen-Chung Chen, Junne-Ming Sung, Nobuyo Maeda, Pei-Jane Tsai

**Affiliations:** 1 Institute of Basic Medical Sciences, National Cheng Kung University, Tainan 70101, Taiwan; 2 Institute of Clinical Medicine, National Cheng Kung University, Tainan 70101, Taiwan; 3 Research Center of Clinical Medicine, National Cheng Kung University Hospital, Tainan 70403, Taiwan; 4 Department of Physiology, National Cheng Kung University, Tainan 70101, Taiwan; 5 Institute of Molecular Biology, Academia Sinica, Taipei 11529, Taiwan; 6 Institute of Microbiology and Immunology, Chung Shan Medical University, Taichung 40201, Taiwan; 7 National Laboratory Animal Center, National Applied Research Laboratories, Taipei 11529, Taiwan; 8 Department of Medical Laboratory Science and Biotechnology, National Cheng Kung University, Tainan 70101, Taiwan; 9 Department of Pathology, National Cheng Kung University Hospital, Tainan 70403, Taiwan; 10 Division of Nephrology, Department of Internal Medicine, National Cheng Kung University Hospital, Tainan 70403, Taiwan; 11 Department of Pathology and Laboratory Medicine, University of North Carolina at Chapel Hill, North Carolina 27599, USA; 12 Research Center of Infectious Disease and Signaling, National Cheng Kung University, Tainan 70101, Taiwan

## Abstract

PPARγ modulates energy metabolism and inflammation. However, its specific functions in the balance of immunity *in vivo* have been explored incompletely. In this study, by the age of 14 mo, *Pparg*^*C/−*^ mice with PPARγ expression at 25% of the normal level exhibited high autoantibody levels and developed mesangial proliferative glomerulonephritis, which resembled systemic lupus erythematosus (SLE)-like autoimmune disease. These symptoms were preceded by splenomegaly at an early age, which was associated with increases in splenocyte accumulation and B-cell activation but not with relocation of hematopoiesis to the spleen. The mechanism of splenic lymphocyte accumulation involved reduced sphingosine-1-phosphate receptor 1 (S1P_1_) expression and diminished migration toward S1P in the *Pparg*^*C/*−^ splenocytes, which impeded lymphocyte egression. Mechanistically, increased Th17 polarization and IL-17 signaling in the *Pparg*^*C/−*^ CD4^+^ T cells contributed to B-cell hyperactivation in the spleen. Finally, the activation of the remaining PPARγ in *Pparg*^*C/−*^ mice by pioglitazone increased S1P_1_ levels, reduced the Th17 population in the spleen, and ameliorated splenomegaly. Taken together, our data demonstrated that reduction of *Pparg* expression in T-helper cells is critical for spontaneous SLE-like autoimmune disease development; we also revealed a novel function of PPARγ in lymphocyte trafficking and cross talk between Th17 and B cells.

Over several decades, the prevalence of metabolic and autoimmune diseases has increased in Western countries^1^,^2^. Notably, arthritis (referring to more than 100 rheumatic diseases) and obesity maps have shown considerable overlaps (http://www.cdc.gov/obesity/data/prevalence-maps.html and http://www.cdc.gov/arthritis/data_statistics/state-data-current.htm). Furthermore, familial partial lipodystrophy, a type of body fat loss, is associated with autoimmune diseases^3^,^4^. The association between dysregulated metabolic balance and autoimmune diseases suggests that common etiological factors underlie both conditions^5^. We hypothesize that peroxisome proliferator-activated receptor gamma (PPARγ) is one of these factors.

PPARγ is a transcription factor involved in adipocyte differentiation and glucose metabolism. It has also been implicated in modulating inflammation and immune responses. Among cell-specific knockout mouse models, PPARγ CD4^+^ T-cell–specific knockout mice have enhanced T-helper 17 (Th17) differentiation and are more susceptible to myelin oligodendrocyte glycoprotein (MOG)-induced experimental allergic encephalomyelitis (EAE)^6^. Macrophage-specific PPARγ knockout mice develop systemic lupus erythematosus (SLE) nephritis caused by deficient phagocytosis^7^. Among haploinsufficient mouse models, *Pparg*^+*/−*^ B cells show increased proliferation, and *Pparg*^+*/−*^ mice are more susceptible to ovalbumin or methylated BSA-induced arthritis^8^. By contrast, *Pparg*^+*/−*^ mice are susceptible to MOG-induced EAE, which is associated with an increase in T-cell proliferation and Th1 response^9^. Thus, PPARγ loss implicates the susceptibility of an individual to autoimmunity.

Because the influence of individual genes on autoimmune disease development involves multiple regulatory pathways, the conclusions obtained using cell-type–specific knockout models may be somewhat biased. Although most of the aforementioned studies were conducted in a cell-specific or haploinsufficient manner with the stimulation of specific antigens, the detailed regulation of the balance between tolerance and immunity by PPARγ might have been masked in those experimentally induced systems. Furthermore, subtle gene expression variations have been linked to autoimmune disease development in mouse models^10^,^11^. Moreover, clinical studies have shown that single-nucleotide polymorphisms, manifested as a modest change in gene expression, are often associated with autoimmunity^12^,^13^. Thus, a modest change in gene expression could shift the balance between tolerance and autoimmunity. A novel tool for revealing the actual functions of PPARγ in the development of autoimmunity without stimulating specific antigens is required.

In this study, we investigated the function of PPARγ in the humoral immune response by using mice with different levels of PPARγ expression (25%–100%) to titrate the PPARγ dose effects on the immune system. These PPARγ quantitative variant mouse strains differ only in the *Pparg* 3′-UTR sequence and produce normal PPARγ protein in all relevant tissues^14^,^15^,^16^. Thus, these PPARγ quantitative variants are useful for revealing the involvement of PPARγ in the complex immune system. Here, we reported that young mice with PPARγ expression at 25% of the normal level showed splenomegaly independent of extramedullary hematopoiesis compared with mice with ≥50% PPARγ expression. Because the disturbance and hyperactivation of the immune system are frequently associated with splenomegaly, we hypothesized that immunological homeostasis is disrupted at a certain low level of PPARγ expression, consequently enhancing humoral responses and resulting in autoimmunity.

## Results

### Spleen enlargement in PPARγ hypomorphic mice

**Figure 1:**
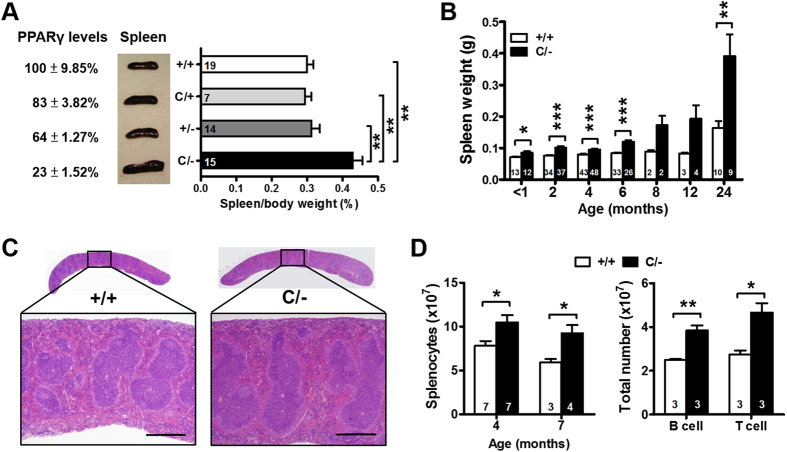
Spleen enlargement in PPARγ hypomorphic mice. (**A**) Percentage of splenic PPARγ expression, gross morphology of spleens, and spleen-to-body weight ratio from WT, *Pparg*^*C/*+^, *Pparg*^+*/−*^ and *Pparg*^*C/−*^ mice at 2–3 mo of age by one-way ANOVA with Scheffe’s test. (**B**) Spleen weight at different ages. (**C**) Hematoxylin and eosin staining of spleen sections from 3-mo-old WT and *Pparg*^*C/−*^ mice. Scale bar, 500 μm. (**D**) Total splenic cellularity of 4- and 7-mo-old mice, and total B cells and T cells in the spleens of 4-mo-old mice. Numbers inside bars indicate the number for each group. *p < 0.05; **p < 0.01; ***p < 0.001.

Four mouse strains, *Pparg*^*C/−*^, *Pparg*^+*/−*^, *Pparg*^*C/*+^, and *Pparg*^+*/*+^ (wild-type [WT]), with 23%, 64%, 83%, and 100% PPARγ expression in the spleen were generated by crossing *Pparg*^+*/−*^ mice with *Pparg*^*C/*+^ mice, which carry an allele of *Pparg* with a *c-fos* AU-rich element inserted in the 3′-UTR region (Fig. 1A)^14^,^15^. The reduction was confirmed by the immunofluorescent staining without the change of cellular localization (Fig. S1A). In addition, the ratio to PPARγ level of Ser273 phosphorylation, which is known to inhibit its transactivation^17^, was higher in *Pparg*^*C/−*^ splenocytes (Fig. S1B), suggesting that PPARγ activity may be even repressed in *Pparg*^*C/−*^ splenocytes. At 2–3 mo of age, among all littermates, only *Pparg*^*C/−*^ mice exhibited splenomegaly (Fig. 1A). The increase in spleen weight in *Pparg*^*C/−*^ mice compared with WT littermates occurred at all ages beginning at 1 mo in mice of both sexes, and the differences became highly apparent after the age of 12 mo (Figs 1B and S1C,D). *Pparg*^*C/−*^ mice aged 4 mo showed a normal splenic architecture (Fig. 1C) but increased numbers of total splenocytes, B cells, and T cells (Fig. 1D). However, the composition of immune cell populations in the spleen**—**including B and T cells and their subsets as well as dendritic cells (CD11C^+^FSC^+^), macrophages (Gr-1^−^CD11b^+^F4/80^+^), plasma cells (CD19^low^CD138^+^), germinal center B cells (B220^+^PNA^high^), and activated CD4^+^ T cells (CD62L^high^CD44^high^)—did not differ between WT and *Pparg*^*C/−*^ mice at both ages (Tables SI–II). Thus, splenomegaly in *Pparg*^*C/−*^ mice was associated with the increase in total cellularity, rather than with a specific increase in a certain subpopulation.

### No signs of extramedullary hematopoiesis in young *Pparg* ^*C/−*^ mice

To investigate whether extramedullary hematopoiesis causes splenomegaly in *Pparg*^*C/−*^ mice, we performed microcomputed tomography analysis on the distal metaphysis of femurs harvested from younger (2-mo-old) and older (9-mo-old) mice (Fig. S2A). Compared with their WT littermates, older *Pparg*^*C/−*^ mice exhibited significantly increased bone mineral density, trabecular volumes, and trabecular numbers but decreased trabecular separation (Fig. S2B); however, these parameters did not differ among the younger mice. Furthermore, the percentage of Lin^−^cKit^+^Sca1^+^ hematopoietic stem cells (HSCs) in the spleen of the older *Pparg*^*C/−*^ mice was significantly higher than that in their WT littermates (Fig. S2C). The expression of transcription factors crucial for HSC differentiation and self-renewal—LMO2 and GATA1—was significantly higher in the spleen but lower in the bone marrow of the older *Pparg*^*C/−*^ mice (Fig. S2D); however, these parameters did not differ between the younger WT and *Pparg*^*C/−*^ mice. These data demonstrate that splenomegaly is independent of extramedullary hematopoiesis in younger *Pparg*^*C/−*^ mice.

### Reduced sphingosine-1-phosphate receptor 1 expression and migration in the splenocytes of PPARγ hypomorphic mice

**Figure 2:**
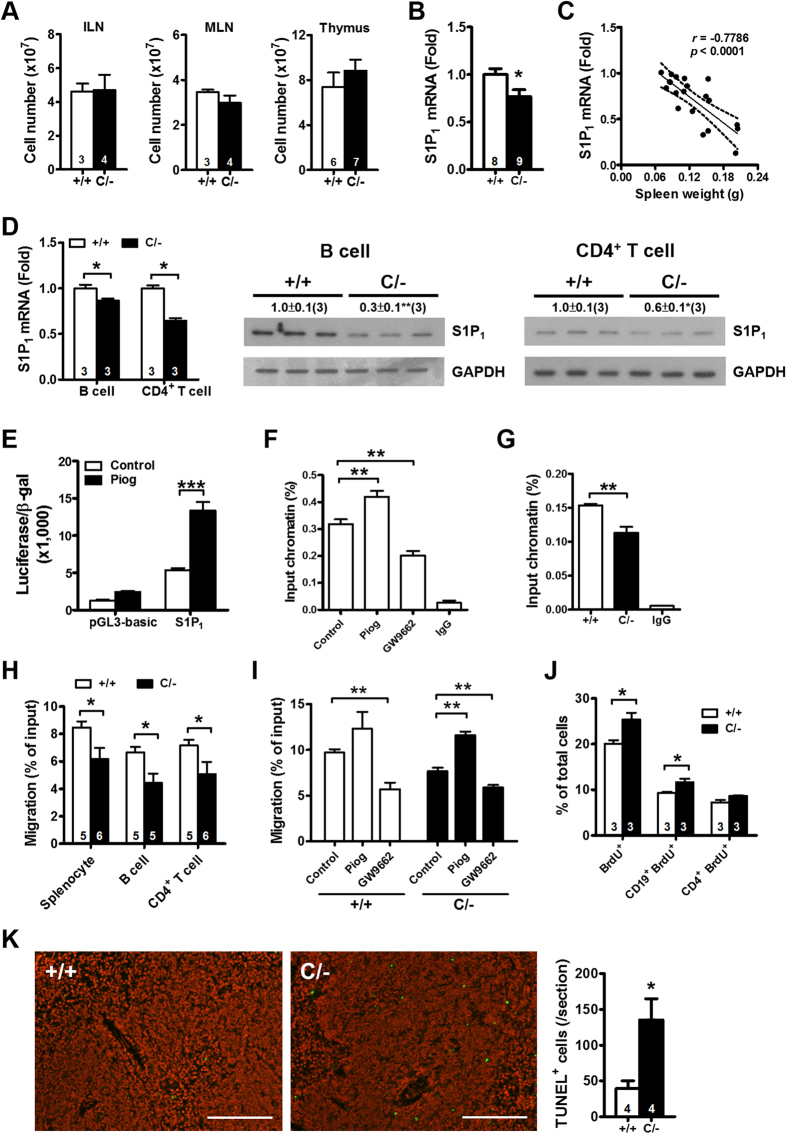
Cell accumulation and proliferation in the spleen of PPARγ hypomorphic mice. (**A**) Total cellularity of inguinal (ILN) and mesenteric (MLN) lymph nodes of 3-mo-old mice and thymus of 6-wks-old mice. (**B**) S1P_1_ mRNA level in the spleen of 3-mo-old mice. (**C**) Relationship between spleen weight and S1P_1_ mRNA level. (**D**) S1P_1_ mRNA and protein levels in B cells and CD4^+^ T cells from 3-mo-old mice. (**E**) Promoter activity of HEK293T cells transiently transfected with reporter constructs and treated with or without 80 μM pioglitazone (Piog) for 24 hours. (**F**) ChIP-PCR in WT splenocytes with Piog or GW9662 treatment for 24 hours. (**G**) ChIP-PCR in WT and *Pparg*^*C/−*^ splenocytes. Sequences containing the potential PPARγ binding site in *S1P*_*1*_ were amplified by real-time PCR. (**H**) Migration of splenocytes, B cells and CD4^+^ T cells in response to 100 nM S1P in Transwell migration assays. (**I**) Migration of splenocytes in response to 200 nM S1P after 24-hour treatment with 40 μM pioglitazone or 40 μM GW9662. (**J**) Percentages of cells incorporating BrdU for all lymphocytes, CD19^+^ B cells, and CD4^+^ T cells of 3-mo-old mice. (**K**) Apoptotic cells (*green*) in the spleen of 3-mo-old mice detected by a TUNEL assay. Scale bar, 150 μm. Numbers inside bars or parentheses indicate the number for each group. *p < 0.05; **p < 0.01; ***p < 0.001.

We next examined whether other immune organs of younger *Pparg*^*C/−*^ mice also demonstrated increased cellularity. The inguinal and mesenteric lymph nodes of 3-mo-old mice and the thymus of 6-wk-old *Pparg*^*C/−*^ mice showed normal cellularity (Fig. 2A). Moreover, the numbers of circulating leukocytes, lymphocytes, B cells (CD19^+^), and T cells (CD3^+^) were similar between WT and *Pparg*^*C/−*^ mice (Table SIII). The normal cellularity of leukocytes in blood and of the immune organs (except the spleen) suggested that the increase in the number of splenocytes in younger *Pparg*^*C/−*^ mice is caused by a trafficking defect.

We first examined the expression of CXCR4 and CXCR5, which are crucial in lymphocytes homing to central lymphoid tissues^18^, on B cells and CD4^+^ T cells, and found none of them were different (Fig. S3A). The predominant lysophopholipid sphingosine-1-phosphate (S1P) receptor, S1P_1_, is essential for T- and B-cell egression from peripheral lymphoid organs^19^,^20^. Compared with WT mice, *Pparg*^*C/−*^ mice exhibited a decreased S1P_1_ mRNA level in the spleen (Fig. 2B). Furthermore, S1P_1_ expression levels were negatively correlated with spleen weight in *Pparg*^*C/−*^ mice (Fig. 2C). Splenic B cells and CD4^+^ T cells of *Pparg*^*C/−*^ mice also showed reduced S1P_1_ mRNA and protein levels (Fig. 2D). We performed experiments to confirm that PPARγ acts as a transcription factor in regulation of S1P_1_ expression. In both WT and *Pparg*^*C/−*^ splenocytes, pioglitazone (PPARγ agonist) and GW9662 (PPARγ antagonist) increased and reduced S1P_1_ levels, respectively (Fig. S3B). To further ascertain whether S1P_1_ expression is directly regulated by PPARγ, the *S1p1* promoter and its adjacent sequences were analyzed in silico by using the MatInspector tool. A putative PPARγ responsive element (PPRE) was predicted downstream of the transcription start site at +16 to +38 (score: 0.898) of *S1p1*. We cloned this region for a reporter assay and observed that its luciferase activity was significantly enhanced by pioglitazone (Fig. 2E). Chromatin immunoprecipitation-PCR in WT splenocytes showed that pioglitazone increased the binding of PPARγ to this predicted PPRE in *S1p1*, whereas GW9662 abrogated it (Fig. 2F). Finally, *Pparg*^*C/−*^ splenocytes showed lesser binding of PPARγ to *S1p1* than WT splenocytes did (Fig. 2G). These results suggest that PPARγ-mediated S1P_1_ expression involves direct transcriptional regulation.

We then studied the function of PPARγ in splenocyte migration toward S1P by using a Transwell assay. *Pparg*^*C/−*^ splenocytes, B cells, and CD4^+^ T cells showed significantly impaired migration toward S1P (Fig. 2H), and they lost their dose-dependent effect in response to the increasing dose of S1P (Fig. S3C–E). Pioglitazone increased migration in both WT and *Pparg*^*C/−*^ splenocytes, whereas GW9662 reduced it (Fig. 2I). Finally, the treatment of mouse embryonic fibroblasts with GW9662 downregulated the S1P_1_ levels (Fig. S3F). These data suggested that PPARγ regulates splenocyte migration, which is correlated with S1P_1_ levels. Therefore, the decreased S1P_1_ expression in the *Pparg*^*C/−*^ splenocytes likely reduces their egression from the spleen, resulting in their splenic accumulation.

### Increased B-cell proliferation and apoptotic cells in the spleen of PPARγ hypomorphic mice *in vivo*

The increased cellularity of the spleen may also be associated with alterations in cell proliferation and apoptosis. An *in vivo* BrdU incorporation assay showed that the proliferation of total splenocytes and CD19^+^ B cells , but not CD4^+^ T cells, increased in *Pparg*^*C/−*^ mice compared with that in WT mice (Fig. 2J). However, the *in vitro* proliferation of B cells and CD4^+^ T cells, in response to various stimulations, was similar between WT and *Pparg*^*C/−*^ mice (Fig. S4A,B). A TUNEL assay demonstrated more TUNEL-positive cells in the spleen of *Pparg*^*C/−*^ mice than in that of WT mice (Fig. 2K). However, the apoptosis rates of B cells and CD4^+^ T cells did not differ in culture between the *Pparg*^*C/−*^ and WT mice (Fig. S4C,D). These results suggest that the increased B-cell proliferation and apoptosis *in vivo* are attributable to the microenvironment of the *Pparg*^*C/−*^ spleen.

### Increased autoantibody production and SLE-like autoimmune disease development in older PPARγ hypomorphic mice

**Figure 3:**
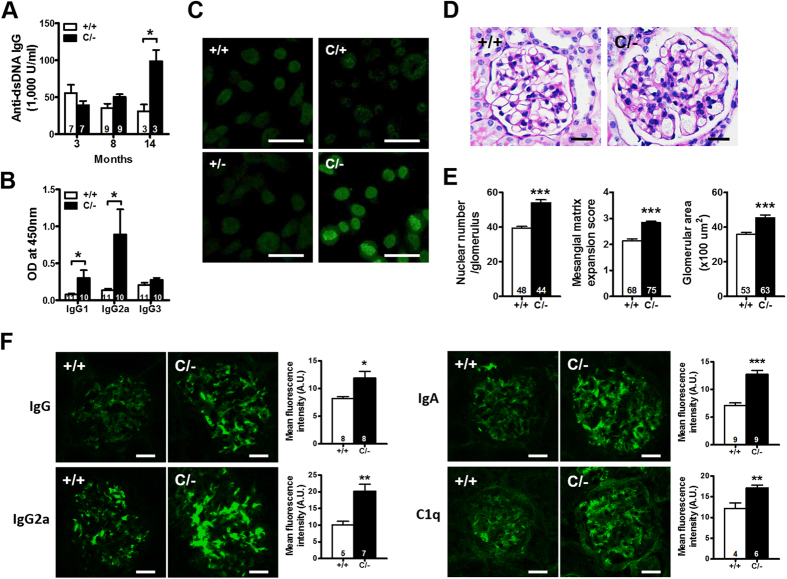
The development of autoimmune disorders in older PPARγ hypomorphic mice. (**A**) Antibodies against dsDNA in the sera from mice at different ages. (**B**) IgG subclass of anti-dsDNA in the sera of 14-mo-old mice. (**C**) HEp-2 cells stained for anti-nuclear antibodies from the sera of 14-mo-old mice. Scale bar, 40 μm. (**D**) Representative periodic acid-Schiff (PAS)-stained glomerular morphology and (**E**) histomorphometric quantification of 14-mo-old mice. Scale bar, 20 μm. (**F**) Representative immunofluorescent images and quantifications of immune complex deposition for IgG, IgG2a, IgA and C1q in the glomeruli of 14-mo-old mice. Scale bar, 20 μm. Relative mean fluorescence intensity was analyzed using ImageJ software. Numbers inside bars indicate the number for each group. *p < 0.05; **p < 0.01; ***p < 0.001.

We next investigated the autoimmune status of *Pparg*^*C/−*^ mice. The serum levels of antibodies against dsDNA did not differ between *Pparg*^*C/−*^ and WT mice aged ≤8 mo. However, the sera from 14-mo-old *Pparg*^*C/−*^ mice showed significantly higher levels of antibodies against dsDNA (Fig. 3A), with significant increases in the levels of IgG1 and IgG2a (Fig. 3B). The sera of the older *Pparg*^*C/−*^ mice also showed detectable levels of antibodies against nuclei (Fig. 3C). Increased autoantibody production probably mediates the pathogenesis of autoimmune disease; for example, it likely induces SLE. Accordingly, 14-mo-old *Pparg*^*C/−*^ mice showed mesangial proliferative glomerulonephritis, characterized by glomerular cell proliferation, mesangial matrix expansion, and enlarged glomeruli (Fig. 3D,E), despite normal urine albumin levels, daily urinary albumin excretion, and serum creatinine levels (Fig. S5A). Immunofluorescent staining of kidney tissue revealed granular deposition of IgG, IgG2a, IgA, and C1q in the glomerular mesangium of the older *Pparg*^*C/−*^ mice (Fig. 3F). Electron microscopic examination revealed electron-dense deposits in the expanded mesangium without involvement of the glomerular capillary loops (Fig. S5B). These results demonstrate that nuclear antigen-specific autoantibodies, immune complex deposition, and glomerular injury in older *Pparg*^*C/−*^ mice, resembling SLE-like autoimmune diseases.

### PPARγ deficiency in effector T-helper cells increases B-cell activation

**Figure 4:**
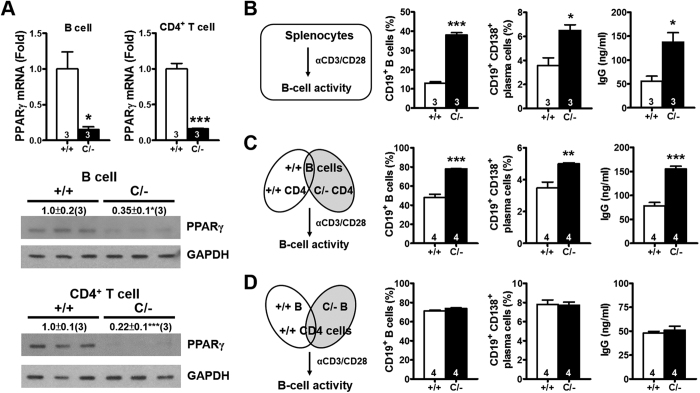
Increased B-cell activation by PPARγ hypomorphic effector T-helper cells. (**A**) PPARγ mRNA and protein levels in B cells and CD4^+^ T cells from 3-mo-old mice. (**B**) Schematic of the splenocyte activation protocol. Percentages of B cells (CD19^+^) gating from lymphocytes and proliferating blast cells, percentages of plasma cells (CD19^+^ CD138^+^) gating from B cells (CD19^+^) measured by flow cytometry after anti-CD3/CD28 stimulation for 3 d, and IgG production in the medium measured after anti-CD3/CD28 stimulation for 7 d. (**C**) Coculture of WT B cells with WT or *Pparg*^*C/−*^ CD4^+^ T cells, the same parameters as in B were measured. (**D**) Coculture of WT CD4^+^ T cells with WT or *Pparg*^*C/−*^ B cells, the same parameters as in B were measured. Splenocytes, B cells and CD4^+^ T cells were isolated from 4–6-mo-old mice. Numbers inside bars or parentheses indicate the number for each group. *p < 0.05; **p < 0.01; ***p < 0.001.

Because B-cell proliferation and activation are associated with increased autoantibody production, we next tested whether the increased *Pparg*^*C/−*^ B-cell proliferation *in vivo* is associated with nearby PPARγ hypomorphic CD4^+^ T cells. Both mRNA and protein levels of PPARγ in the B cells and CD4^+^ T cells from the *Pparg*^*C/−*^ spleen were significantly lower (Fig. 4A), confirming the presence of the PPARγ hypomorph in both cell types. Because the *Pparg*^*C/−*^ spleen showed normal lymphocyte composition, equal numbers of WT and *Pparg*^*C/−*^ splenocytes were treated with anti-CD3/CD28 antibodies for comparing T-cell activation (Fig. 4B). After 3 d, the percentages of B cells and plasma cells in the *Pparg*^*C/−*^ splenocyte culture increased considerably (Fig. 4B). Moreover, after 7 d, the IgG levels in the *Pparg*^*C/−*^ splenocyte culture medium were significantly higher than those in the WT splenocyte culture medium.

To further analyze the contribution of B cells and CD4^+^ T cells in B-cell hyperactivation in *Pparg*^*C/−*^ splenocytes, we isolated B cells and CD4^+^ T cells from *Pparg*^*C/−*^ and WT spleens to perform coculture experiments. A WT B-cell and *Pparg*^*C/−*^ CD4^+^ T-cell coculture generated higher percentages of B cells and plasma cells and higher levels of secreted IgG than did a WT B-cell and WT CD4^+^ T-cell coculture (Fig. 4C). By contrast, the activities of *Pparg*^*C/−*^ B cells and WT B cells cocultured with WT CD4^+^ T cells did not differ (Fig. 4D). Moreover, a *Pparg*^*C/−*^ B-cell and *Pparg*^*C/−*^ CD4^+^ T-cell coculture generated higher percentages of plasma cells and higher levels of secreted IgG than a *Pparg*^*C/−*^ B-cell and WT CD4^+^ T-cell coculture did (Fig. S6A). By contrast, the activities of *Pparg*^*C/−*^ B cells and WT B cells cocultured with *Pparg*^*C/−*^ CD4^+^ T cells did not differ (Fig. S6B).

We further elucidated the effect of the PPARγ hypomorph on B cells by examining the responses of *Pparg*^*C/−*^ B cells to T-cell independent (T-I) and dependent (T-D) antigens *in vitro*. Splenic B cells from *Pparg*^*C/−*^ and WT mice were stimulated with the T-I antigens LPS (T-I type I) and NP-Ficoll (T-I type II), or anti-CD40 plus IL-4 (mimic T-D antigen). Upon stimulation with both T-I antigens, *Pparg*^*C/−*^ B cells produced IgM and IgG levels similar to those of WT B cells (Fig. S6C). Furthermore, *Pparg*^*C/−*^ B cells showed a normal response mimicking T-D antigen stimulation for IgM and IgG production (Fig. S6D). *Pparg*^*C/−*^ B-cells also showed normal expression of Blimp-1 and Bcl-6, which are important for induction of plasma cells and germinal center reaction^21^,^22^, in response to NP-Ficoll and anti-CD40 plus IL-4 stimulation (Fig. S6E). These results suggested that *Pparg*^*C/−*^ B cells have normal intrinsic responses to mitogen and costimulatory signals. Thus, the increased B-cell activity in the *Pparg*^*C/−*^ spleen is likely caused by the presence of PPARγ hypomorphic effector T-helper cells, rather than reduced PPARγ expression in B cells.

### Increased Th17 polarization in PPARγ hypomorphic mice

**Figure 5:**
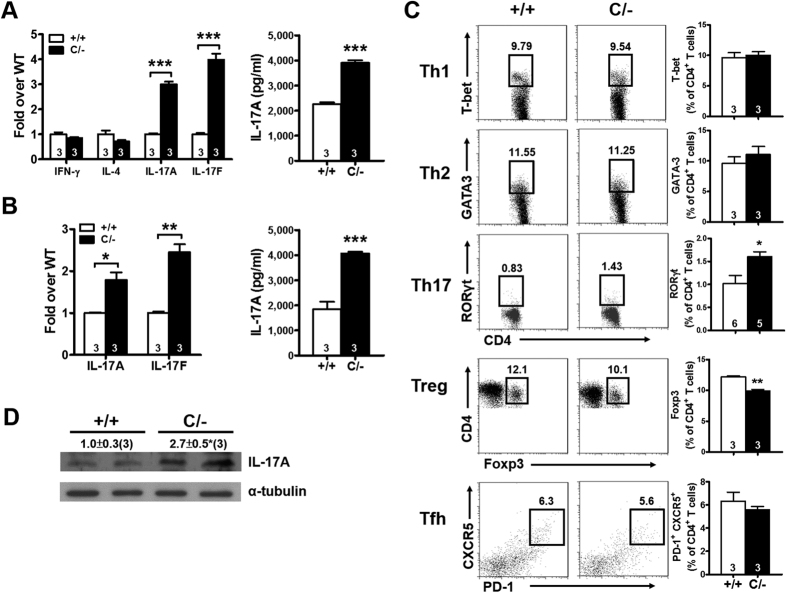
Increased Th17 population in the spleen of PPARγ hypomorphic mice. (**A**) Relative expression of cytokines in the splenic CD4^+^ T cells and the protein levels of IL-17A in the medium stimulated with anti-CD3/CD28 for 3 d. (**B**) Relative expression of IL-17 in the splenic CD4^+^ T cells and the protein levels of IL-17A in the medium stimulated with TGF-β (5 ng/ml) and IL-6 (20 ng/ml) for 3 d. (**C**) Flow cytometric analysis of transcription factors (T-bet, GATA-3, RORγt, and Foxp3) in the splenic CD4^+^ T cells and expression of PD-1 and CXCR5 on the splenic CD4^+^ T cells. (**D**) Immunoblotting of IL-17A in the spleen. Mice are 4–6-mo-old. Numbers inside bars or parentheses indicate the number for each group. *p < 0.05; **p < 0.01; ***p < 0.001.

To determine the type of effector CD4^+^ T cell in the *Pparg*^*C/−*^ spleen that affects B cell activation, we stimulated splenic CD4^+^ T cells *in vitro* by using anti-CD3/CD28 antibodies and assessed T-helper cell differentiation according to signature cytokine production. Although the mRNA levels of IFN-γ and IL-4 did not differ between WT and *Pparg*^*C/−*^ CD4^+^ T cells, those of IL-17A and IL-17F were considerably higher in the stimulated *Pparg*^*C/−*^ CD4^+^ T cells (Fig. 5A). Similarly, IL-17A levels in the cultured medium of stimulated *Pparg*^*C/−*^ CD4^+^ T-cells was significantly higher. These results suggested that *Pparg*^*C/−*^ CD4^+^ T cells exhibit a greater capacity for Th17 polarization in response to anti-CD3/CD28 stimulation than WT cells do. Furthermore, the *in vitro* stimulation of *Pparg*^*C/−*^ CD4^+^ T cells under Th17 differentiation significantly increased the mRNA levels of IL-17A and IL-17F as well as the protein level of IL-17A (Fig. 5B). Furthermore, *Pparg*^*C/−*^ CD4^+^ T cells showed significantly higher levels of the transcription factor RORγt and lower levels of Foxp3 compared with WT CD4^+^ T cells, whereas the levels of T-bet and GATA-3 in CD4^+^ T cells were similar in both genotypes (Fig. 5C). Although Park *et al.* reported that PPARγ CD4^+^ T-cell–specific knockout mice have an increased number of follicular T-helper (Tfh) cells^23^, we observed that the number of Tfh cells, stained using PD-1 and CXCR5, was not altered in both young and older *Pparg*^*C/−*^ mice (Figs 5C and S7). We also observed increased IL-17A protein levels in the spleen of *Pparg*^*C/−*^ mice (Fig. 5D). These results indicate that Th17 polarization increases in the *Pparg*^*C/−*^ spleen, and this increase is correlated with B-cell hyperactivation.

### Enhanced Th17 function promotes B-cell activation through IL-17 signaling in PPARγ hypomorphic mice

**Figure 6:**
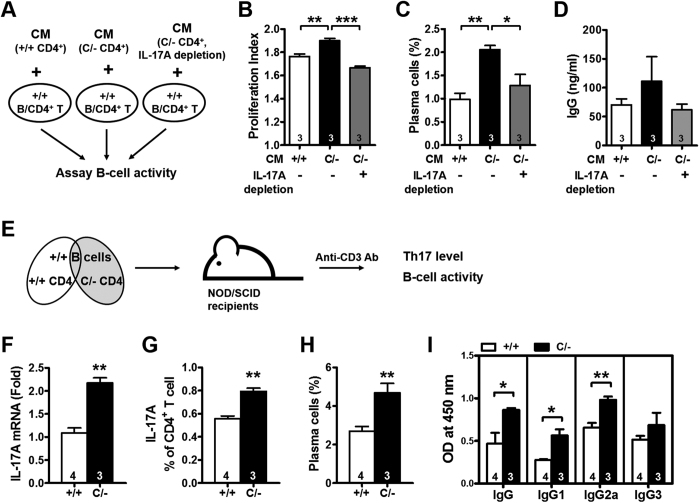
Increased B-cell activation by the IL-17 secreted from PPARγ hypomorphic CD4^+^ T cells. (**A**) Schematic of the WT B-cell activation protocol. Incubation of B cells and CD4^+^ T cells (both from WT mice) with the conditioned medium (CM) from anti-CD3/CD28-activated WT and *Pparg*^*C/−*^ CD4^+^ T cells with or without IL-17A depletion. (**B**) B-cell proliferation was analyzed by flow cytometry and the proliferation index was quantified by FlowJo software. (**C**) Percentage of plasma cells and (**D**) IgG production were determined. B cells and CD4^+^ T cells were isolated from 4–6-mo-old mice. *p < 0.05; **p < 0.01; ***p < 0.001 by one-way ANOVA with Scheffe’s test for (**B**–**D**). (**E**) Schematic of the adoptive transfer protocol. WT or *Pparg*^*C/−*^ CD4^+^ T cells were mixed with WT B cells and transferred into NOD/SCID mice. The recipient mice were stimulated with anti-CD3 antibodies (40 μg/d) for 5 d and analyzed 11 d after the end of stimulation. (**F**) Relative expression of IL-17A in the splenic CD4^+^ T cells. (**G**) Splenic CD4^+^ T cells were assessed for IL-17A production after PMA/inomycin restimulation through intracellular staining. (**H**) Percentage of plasma cells in splenocytes measured by flow cytometry. (**I**) IgG, IgG1, IgG2a and IgG3 levels in the sera. Numbers inside bars indicate the number for each group. *p < 0.05 and **p < 0.01 by Student’s *t* test for (**F**–**I**).

To determine the relationship between enhanced Th17 polarization and B-cell hyperactivation in *Pparg*^*C/−*^ mice, the conditioned medium from activated WT or *Pparg*^*C/−*^ CD4^+^ T cells was added to the WT B-cell and WT CD4^+^ T-cell coculture (Fig. 6A). The conditioned medium from the activated *Pparg*^*C/−*^ CD4^+^ T cells showed increased B-cell proliferation, plasma cell percentages, and IgG production compared with the medium from the activated WT CD4^+^ T cells (Fig. 6B–D). Depletion of IL-17A by antibodies in the conditioned medium of activated *Pparg*^*C/−*^ CD4^+^ T cells reversed these changes, demonstrating that IL-17A is an effective mediator of increased B-cell activation in *Pparg*^*C/−*^ mice.

To further test these findings *in vivo*, we mixed WT or *Pparg*^*C/−*^ CD4^+^ T cells with WT B cells, transferred them into NOD/SCID mice, which lack T and B cells, and stimulated the mice with anti-CD3 antibodies (Fig. 6E). PPARγ hypomorph in T cells increased IL-17A mRNA and protein levels in CD4^+^ T cells, the percentage of plasma cells in the splenocytes, and levels of circulating IgG, IgG1 and IgG2a (Fig. 6F–I). These results suggested that observed Th17 polarization and lupus like phenotypes in the aged *Pparg*^*C/-*^ mice are modulated by PPARγ in a T cell-intrinsic fashion.

### Pioglitazone ameliorates splenomegaly in PPARγ hypomorphic mice

**Figure 7:**
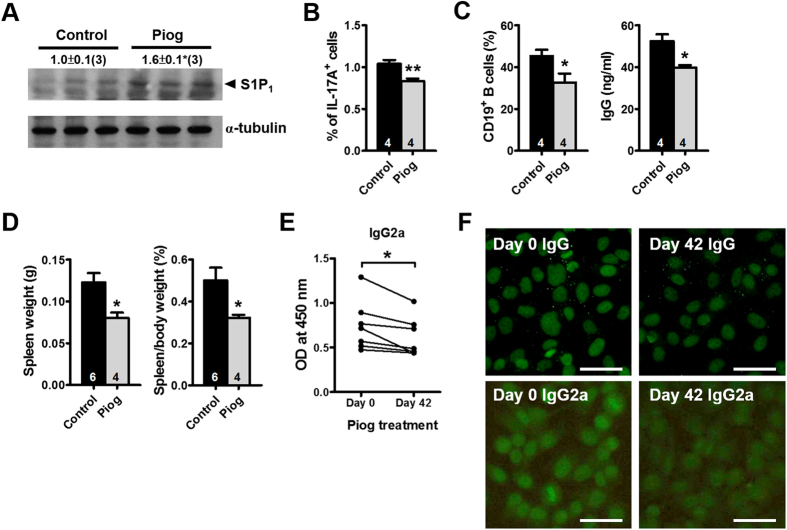
Effects of PPARγ activation in PPARγ hypomorphic mice. (**A**) Immunoblotting of S1P_1_ and (**B**) percentage of IL-17A^+^ CD4^+^ T cells in the spleen of *Pparg*^*C/−*^ mice treated with 20 mg/kg/d pioglitazone (Piog) for 2 mo starting at 2 mo of age. (**C**) Percentage of CD19^+^ B cells and secreted IgG levels from the coculture of WT B-cells with CD4^+^ T cells from control and Piog-treated *Pparg*^*C/−*^ mice. (**D**) Spleen weight and spleen-to-body weight ratio in Piog-treated *Pparg*^*C/−*^ mice. (**E**) The IgG2a levels in and (**F**) HEp-2 cells stained for anti-nuclear antibodies (IgG and IgG2a) from the sera of *Pparg*^*C/−*^ mice treated with 60 mg/kg/d Piog for 6 wks starting at 14 mo of age. Numbers inside bars or parentheses indicate the number for each group. *p < 0.05 and **p < 0.01.

We further evaluated whether activation of the remaining PPARγ sufficiently reverses the changes in *Pparg*^*C/−*^ mice. Treatment with low-dose pioglitazone for 2 mo starting at 2 mo of age increased S1P_1_ levels and reduced the Th17 population in the spleen of *Pparg*^*C/−*^ mice (Fig. 7A,B). Furthermore, CD4^+^ T cells isolated from pioglitazone-treated *Pparg*^*C/−*^ mice exhibited a lower potential for induction of B-cell activity than those isolated from nontreated control *Pparg*^*C/−*^ mice did (Fig. 7C). Consequently, pioglitazone treatment reversed splenomegaly in *Pparg*^*C/−*^ mice (Fig. 7D). Furthermore, treatment with high-dose pioglitazone for 6 wk significantly reduced serum IgG2a levels of older *Pparg*^*C/−*^ mice (Fig. 7E) and attenuated the levels of both IgG and IgG2a against nuclei (Fig. 7F). These results suggest that the activation of 25% PPARγ expression in *Pparg*^*C/−*^ mice promotes S1P_1_ expression in splenocytes and prevents B-cell hyperactivation by attenuating Th17 polarization; these activities collectively contribute to the decrease in spleen weight and, finally, amelioration of the SLE-like autoimmune disease.

## Discussion

Studies have reported that PPARγ deficiency in either adipocytes or hematopoietic cells reduces bone marrow cellularity and promotes extramedullary hematopoiesis in the spleen, leading to splenomegaly^24^,^25^. By contrast, in the current study, younger PPARγ hypomorphic *Pparg*^*C/−*^ mice developed splenomegaly without changes in the bone architecture or relocation of hematopoiesis to the spleen. In these mice, S1P_1_ expression and migration toward S1P of *Pparg*^*C/−*^ splenocytes were decreased, suggesting that hindered lymphocyte egression is a mechanism for their splenic accumulation. We also observed increased *in vivo* B-cell proliferation in the spleen, which may have also contributed to splenomegaly in the younger *Pparg*^*C/−*^ mice. Consequently, the enhanced B-cell activation occurred partly because of the elevated IL-17 levels resulting from the increased Th17 polarization of *Pparg*^*C/−*^ CD4^+^ T cells and not because of the reduction of PPARγ levels in B cells. These factors, along with an increased number of apoptotic cells in the spleen—suggestive of impaired apoptotic cell clearance—likely contributed to the spontaneous development of SLE-like autoimmune disease in the older *Pparg*^*C/−*^ mice.

S1P has a major function in the immune system through binding with the receptors S1P_1_–S1P_5_^26^,^27^. Among these, S1P_1_ is expressed at the highest levels in lymphocytes and is required for lymphocytes to egress from lymphoid organs^19^. The relatively high levels of S1P in circulation enable lymphocytes egression into blood, which is mediated through S1P_1_^19^,^20^. Studies on kidney mesangial cells have shown that PPARγ agonists increase sphingosine kinase 1 levels, leading to increased intracellular S1P levels^28^. Similarly, in mesangial cells, PPARγ activation also upregulates S1P_1_ expression^29^. However, the involvement of similar S1P_1_ regulation by PPARγ in lymphocyte trafficking has never been explored thus far. In this study, we observed reduced S1P_1_ expression in splenic B cells and CD4^+^ T cells of *Pparg*^*C/−*^ mice, which is caused by reduced binding of PPARγ to the genomic *S1p1* sequence. *Pparg*^*C/−*^ B cells and CD4^+^ T cells showed a significantly attenuated migratory response toward S1P. Moreover, pharmacological activation of PPARγ increased S1P_1_ expression and ameliorated splenomegaly. These findings support the involvement of a lymphocytic PPARγ–S1P_1_ axis in lymphocyte egression from the spleen.

In *Pparg*^*C/−*^ mice, the normal aging process was sufficient to cause SLE-like phenotypes. The function of PPARγ in lymphocyte proliferation and autoimmune diseases has been documented in various mouse models^6^,^7^,^8^,^9^; however, most of these studies have induced autoimmunity experimentally and evaluated only the function of PPARγ within individual immune cell types. Thus, they could not replicate the complex interaction of different immune cell types. Here, we devised an *in vitro* coculture system and demonstrated that B cells cocultured with *Pparg*^*C/−*^, but not WT, CD4^+^ T cells became hyperactive regardless of the *Pparg* genotype of the B cells. In the spleen, B-cell follicles are surrounded by T-cell areas, and activated CD4^+^ T cells move toward the T-B border for helping B-cell activation^30^. Particularly, Th17 cells act as B-cell helpers through the secretion of IL-17^31^. Although PPARγ deficiency promotes Th17 differentiation and CD4^+^ T-cell–mediated autoimmunity development^6^, whether this T-cell imbalance also affects B-cell activation and contributes to B-cell–mediated autoimmunity remains unclear. Consistently, we observed that *Pparg*^*C/−*^ CD4^+^ T cells exhibited increased polarization toward Th17 cells. The removal of IL-17A from the coculture medium of B cells and CD4^+^ T cells attenuated its ability to increase B-cell activity, demonstrating that the higher IL-17A levels resulting from increased Th17 polarization are essential for increasing B-cell activity in *Pparg*^*C/−*^ mice. Therefore, in our working model, *Pparg*^*C/−*^ spleen creates a microenvironment where sequestered lymphocytes increase cross talk between *Pparg*^*C/−*^ B cells and Th17-polarized CD4^+^ T cells through the secretion of IL-17, resulting in enhanced B-cell activation.

The mechanism by which PPARγ affects the Th17 polarization program and IL-17 production has been explored previously. PPARγ activation negatively regulates *RORγt* transcription by preserving the silencing mediator for retinoid and thyroid hormone receptors (SMRT) corepressor in the RORγt promoter^6^. In addition, PPARγ activation induces *SOCS3* expression and interferes with the STAT3 signaling pathway, which is essential for the transcription of IL-17A, IL-17F, and RORγt^32^. Therefore, PPARγ antagonism may increase Th17 polarization and IL-17 production through direct induction of RORγt or indirect induction of STAT3. These studies showed that PPARγ activation by an agonist suppressed IL-17 expression. By contrast, after blockade by using an antagonist, PPARγ loses the ability to conduct SOCS3 on inhibition of RORγt expression and IL-17 secretion^32^. Furthermore, treatment with a PPARγ antagonist restores expression of RORγt and IL-17^33^. The findings together indicate that Th17 polarization and IL-17 production are negatively regulated by PPARγ in an indirect fashion.

SLE pathogenesis is complex, involving dysregulation of multiple arms of the immune system, including defective lymphocyte trafficking, B-cell activation, and impaired apoptotic cell clearance^34^,^35^,^36^. Although our results suggested that increased Th17 and B-cell cross talk and its consequent overproduction of autoantibodies causes SLE-like autoimmune disease development in older *Pparg*^*C/−*^ mice, increases in the number of apoptotic cells in the spleen cannot be neglected. Rőszer *et al.* demonstrated that mice with macrophage-specific deletion of PPARγ develop SLE nephritis because of impaired apoptotic cell clearance^7^; although their mice developed severe nephritis at an early age (4–6 mo), they showed normal immune cell expansion and B-cell activity without splenomegaly. Moreover, the role of macrophage activation in SLE pathogenesis has been proposed^37^, and our results showed that the basal and LPS-stimulated expression of IL-1β, iNOS and MCP-1 was higher in *Pparg*^*C/−*^ peritoneal macrophages (Fig. S8). Therefore, the SLE-like autoimmune diseases developed in the older *Pparg*^*C/−*^ mice were likely caused by a combination of symptoms associated with immune dysregulations.

We noticed that the symptoms of SLE-like nephritis in older *Pparg*^*C/−*^ mice are relatively mild. Based on the classification by the International Society of Nephrology and the Renal Pathology Society (ISN/RPS)^38^, we speculated that the glomerulonephritis of older *Pparg*^*C/−*^ mice is morphologically compatible with Class II lupus nephritis. Under electron microscope, we did not find effacement of podocyte foot processes in older *Pparg*^*C/−*^ mice (Fig. S5C), which is consistent with the absence of proteinuria and normal serum creatinine.

Our current results showed some discrepancies with those reported previously. For example, Setoguchi *et al.* reported that PPARγ haploinsufficiency enhances the proliferative response of isolated *Pparg*^+*/−*^ B cells, but not T cells, in culture. By contrast, we observed that the *in vitro* proliferation of both *Pparg*^*C/−*^ B cells and CD4^+^ T cells as well as *Pparg*^+*/−*^ spleen weight were normal. Although the reasons for this discrepancy remain unclear, it may be attributable to subtle differences in the genetic background: our mice were from the F1 generation of crossed 129S6 and C57BL6/J mice, whereas those used by Setoguchi *et al.* were from an ICR outbred background. Notably, although both 129 and C57BL/6 are nonautoimmune strains, a certain combination of their genomes can contribute to the expression of autoimmune phenotypes^39^,^40^. Thus, complex contributions of genetic background could not be disregarded in comparison of different sets of experiments.

PPARγ CD4^+^ T-cell specific knockout mice with nearly no detectable PPARγ protein in CD4^+^ T cells have increased Tfh cells, as well as other helper T-cell subsets, such as Th1, Th2 and Th17^23^. However, in our study, *Pparg*^*C/−*^ mice with PPARγ hypomorph in CD4^+^ T cells showed increased Th17 polarization without altering the development of Th1, Th2 and Tfh cells. The discrepancy between two studies may stem from the PPARγ level in CD4^+^ T cells. In addition, because *Pparg*^*C/−*^ mice exhibit the reduction of PPARγ levels in all cell types, the contribution of PPARγ hypomorph in the cell types other than CD4^+^ T cells cannot be neglected. Nevertheless, these results suggest that the development of Th17 cells is more sensitive to the PPARγ level.

The changes in the PPARγ expression levels in our series of *Pparg* mutants were within the human physiological range as a consequence of polymorphic variations. Studies on the association between *PPARG* polymorphisms and the severity of autoimmune diseases have revealed mixed results. For example, the most common *PPARG* polymorphism, Pro12Ala, which exhibits decreased transcriptional activity^41^, is associated with several autoimmune diseases such as psoriatic arthritis^42^ and rheumatoid arthritis^43^. However, the polymorphism is also associated with delayed onset of multiple sclerosis^44^ and with protection against Graves orbitopathy^45^. Another common *PPARG* polymorphism, C161T, was associated with the longer survival of Japanese patients with IgA nephropathy^46^. In addition, treatment with PPARγ agonists reduces symptoms of autoimmune diseases^6^,^47^,^48^. Glucocorticoid is widely applied in SLE therapy; compared with the inhibitory effects of glucocorticoids on T cells, B cells are less affected and antibody production is largely preserved after short-term or low-dose glucocorticoid administration^49^,^50^. Notably, Th17 cells may be relatively resistant to the effects of glucocorticoids^51^,^52^. Therefore, in contrast to those of pioglitazone treatment, the effects of glucocorticoid treatment on SLE-like autoimmune diseases may vary. Our study thus highlights pioglitazone as a potential candidate for SLE therapy.

A recent clinical study showed that familial partial lipodystrophic patients with PPARγ E157D mutation, which severely reduces target gene transcription, have an increased risk of autoimmune diseases^4^. Thus, our work exemplifies how clinical observations can be mechanistically dissected through a basic investigation in a murine model. In conclusion, the titrated reduction of *Pparg* gene expression in mice demonstrated that the level of PPARγ required for maintaining normal immunity is 25%–50% of that in the WT. Our study also demonstrated that PPARγ hypomorphism causes excessive B-cell response with the aid of Th17 in the T-cell dependent humoral immune response. Thus, our study defines a novel function of PPARγ in lymphocyte trafficking and in cross talk between Th17 and B cells and indicates that decreased expression of the metabolism-related factor PPARγ is a risk factor for autoimmune disease, particularly in association with SLE and related autoimmune diseases.

## Methods

### Mice

Generation of mice carrying the modified *Pparg* locus has been described^15^. *Pparg*^+*/*+^ (WT) and *Pparg*^*C/−*^ mice were F1 littermates from the mating of *Pparg*^*C/*+^ mice on a C57BL/6J background with *Pparg*^+*/−*^ mice on a 129S6 background^53^. Mice were bred and housed in the animal facility of National Cheng Kung University (NCKU). Mice were treated with pioglitazone (20 mg/kg/d) via oral gavage for 2 mo. NOD/SCID mice were purchased from NCKU Laboratory Animal Center. All animal studies were performed according to protocols approved by the Institutional Animal Care and Use Committee of NCKU.

### Microcomputed tomography

Right femurs of 2-mo-old and 9-mo-old mice were dissected free of soft tissue, fixed in formalin and scanned by a microcomputed tomography scanner (Skyscan 1076), and analyzed using a software (Skyscan NV, Aartselaar, Belgium). Image acquisition was performed at 48 kV and 200 μA, with a 0.6° rotation between frames. Micromorphological information was obtained from image stacks, allowing three-dimensional parameters of bone microstructure to be calculated, including trabecular bone volume, bone mineral density, trabecular number and trabecular thickness. Morphometric parameters measured by a CT-analyzer have been validated on both virtual objects and aluminum foil and wire phantoms.

### Identification of PPRE and promoter activity assays

Mouse *S1p1* promoter and its adjacent sequences were analyzed using a MatInpector tool for prediction of PPARγ-binding sites^54^. The fragment of the mouse *S1p1* 5′-flanking region (−834 to +173 bp) was cloned into pGL3-basic vector containing a luciferase reporter system. HEK293 T cells were transfected with reporter plasmids using Lipofectamine 2000 (Invitrogen, Carlsbad, CA, USA). Subsequently, cells were incubated with pioglitazone (80 μM) for an additional 24 hours, and luciferase activity was measured using a Dual-Luciferase Reporter Assay System (Promega, Madison, WI, USA).

### ChIP assay

The procedure for ChIP was described previously^55^. In brief, PPARγ protein was fixed with DNA by 1% formaldehyde for 10 minutes. Cells were harvested and sonicated to fragment DNA (average size of 200~500 bp). PPARγ antibody (Cell Signaling, Beverly, MA, USA) was used to pull down the PPARγ protein and DNA complexes. The potential PPARγ binding site was amplified using the primers 5′-CGTTTGCCTGGAGAAATACCA-3′ and 5′-GACTGAGCTGCGGAGAGCTT-3′.

### Migration assays

Splenocytes were cultured and allowed to migrate through 5 μm pore size Transwell inserts (Merck Millipore, Darmstadt, Germany). The lower wells contained 50 to 200 nM S1P (Sigma-Aldrich, St. Louis, MO, USA). After 3 hours of incubation, the migrated cell from each well were counted with a microscope or analyzed for B-cells and CD4^+^ T-cells by flow cytometry.

### Histopathology

Kidneys were fixed in 4% paraformaldehyde, and stained with a periodic acid-Schiff reagent. Immune complex deposits were analyzed on cryosections, which were fixed in acetone and incubated with Alexa 488-conjugated antibodies against IgG (Invitrogen, Carlsbad, CA, USA) and IgG2a (Abcam, Cambridge, MA, USA), or CruzFluor 488-conjugated antibodies against IgA and C1q (Santa Cruz, Dallas, TX, USA). Fluorescence intensity was analyzed using ImageJ software. For the analysis of cell ultrastructure, transmission electron microscopy was performed from mouse kidney cortex with a Hitachi 7000 TEM.

### BrdU and CFSE labeling

Mice were intraperitoneally injected with BrdU (4 mg/injection) twice daily for 5 d. Splenocytes were stained with antibodies against BrdU (BD Pharmingen, San Diego, CA, USA). For the carboxyfluorescein succinimidyl ester (CFSE) labeling, splenocytes (5 × 10^6^ cells) were labeled with 5 μM CFSE (Sigma-Aldrich, St. Louis, MO, USA) and then washed.

### Lymphocyte proliferation and IgG production

B cells and CD4^+^ T cells were isolated with anti-mouse CD19^+^ microbeads and anti-mouse CD4^+^ microbeads (Miltenyi Biotec, Bergisch Gladbach, Germany). For mitogen stimulation, B cells were cultured with LPS (10 μg/ml, Sigma-Aldrich, St. Louis, MO, USA), anti-IgM F(ab′)_2_ (30 μg/ml, Jackson Immunoresearch, West Grove, Pennsylvania, USA) or anti-CD40 antibodies (10 μg/ml, BD Bioscience, San Jose, CA, USA), and CD4^+^ T cells were cultured with anti-CD3 (4 μg/ml) and anti-CD28 antibodies (4 μg/ml). In the coculture assay, CFSE-labeled B cells and CD4^+^ T cells were cocultured on microtiter wells pre-coated with anti-CD3/CD28 antibodies. After 3 d of co-culture, the cells were stained with CD19-PE (eBioscience, San Diego, CA, USA) and CD138-APC (BD Bioscience, San Jose, CA, USA) to determine the percentage of plasma cells in CFSE^+^ B cells. After 7 d of coculture, the supernatant was collected to determine IgG production by ELISA (Bethyl Laboratories, Montgomery, TX, USA).

### *In vitro* T-cell differentiation

CD4^+^ T cells were stimulated with anti-CD3/CD28 antibodies (4 μg/ml) for 3 d and analyzed for cytokine expression. For the Th17 differentiation, CD4^+^ T cells were stimulated with TGF-β (5 ng/ml) and IL-6 (20 ng/ml) for 3 d. The levels of secreted IL-17A (eBioscience, San Diego, CA, USA) in the supernatant were determined by ELISA.

### Flow cytometry

For the cell surface staining, cells were labeled with various antibodies (eBioscience, San Diego, CA, USA) and analyzed by a FACS-Calibur instrument (BD Bioscience, San Jose, CA, USA). For the intracellular staining of cytokines, after 5 hours of incubation with PMA (50 ng/ml) and ionomycin (1 μg/ml) in the presence of brefeldin A (10 μg/ml), cells were stained with anti-CD4 antibodies, followed by fixation and permeabilization (eBioscience, San Diego, CA, USA) and intracellular staining with T-bet, GATA-3, and RORγt (eBioscience, San Diego, CA, USA).

### RNA isolation and real-time PCR

Total RNA was extracted using REzol (PROtech, Taipei, Taiwan). mRNA levels were analyzed with real-time quantitative RT-PCR (Applied Biosystems, Foster City, CA) with *β-actin* as the reference gene in each reaction.

### Detection of autoantibodies

The serum levels of autoantibodies against dsDNA were determined by ELISA (Alpha Diagnostic International, San Antonio, TX, USA). For detection of anti-nuclear antibodies, HEp-2 cells were fixed and incubated with the mouse sera at a dilution of 1:200, and followed by Alexa 488-conjugated anti-mouse IgG antibodies (Invitrogen, Carlsbad, CA, USA).

### Western blot analysis

Total proteins (20 μg) was separated by SDS-PAGE, transferred to PVDF membranes, and probed with antibodies against PPARγ (Cell Signaling, Danvers, MA, USA), PPARγ Ser273, IL-17A (Santa Cruz, Dallas, TX, USA), S1P_1_ (Abcam, Cambridge, MA, USA) and α-tubulin (Sigma-Aldrich, St. Louis, MO, USA).

### Adoptive transfer

CD4^+^ T cells from WT or *Pparg*^*C/−*^ mice and B cells from WT mice were mixed at 1:1 ratio. These cells were transferred in NOD/SCID mice (2 × 10^7^ cells/mouse). The recipient mice were stimulated with anti-CD3 antibodies (40 μg/d) for 5 d. Eleven days after the end of stimulation, splenocytes from the recipient mice were isolated for mRNA and flow cytometric analyses. Sera from recipient mice were collected, and IgG, IgG1, IgG2a and IgG3 antibodies were measured by using ELISA.

### Statistical analysis

Values are reported as mean ± SEM. Statistical analyses were conducted by Student’s *t* test or one-way ANOVA followed by Scheffe’s multiple comparison test. Differences were considered to be statistically significant at *P* < 0.05.

## Additional Information

**How to cite this article**: Liu, Y.-H. *et al.* Quantitative PPARγ expression affects the balance between tolerance and immunity. *Sci. Rep.* **6**, 26646; doi: 10.1038/srep26646 (2016).

## Supplementary Material

Supplementary Information
