# Supplementary material for: Quantitative PPARγ expression affects the balance between tolerance and immunity
Source: Sci Rep. 2016 May 25;6:26646. doi: 10.1038/srep26646 (PMC4879582; doi:10.1038/srep26646)
Supplement: Supplementary Information [file srep26646-s1.pdf]

Quantitative PPARγ expression affects the balance between tolerance and immunity

Ya-Hui Liu[1], Yau-Sheng Tsai[1,2,3], Shih-Chieh Lin[4], Nan-Shih Liao[5], Ming-Shiou Jan[6], Chung-Tiang Liang[7],

Shih-Wen Hsu[8], Wen-Chung Chen[9], Junne-Ming Sung[10], Nobuyo Maeda[11], and Pei-Jane Tsai[1,8,12]

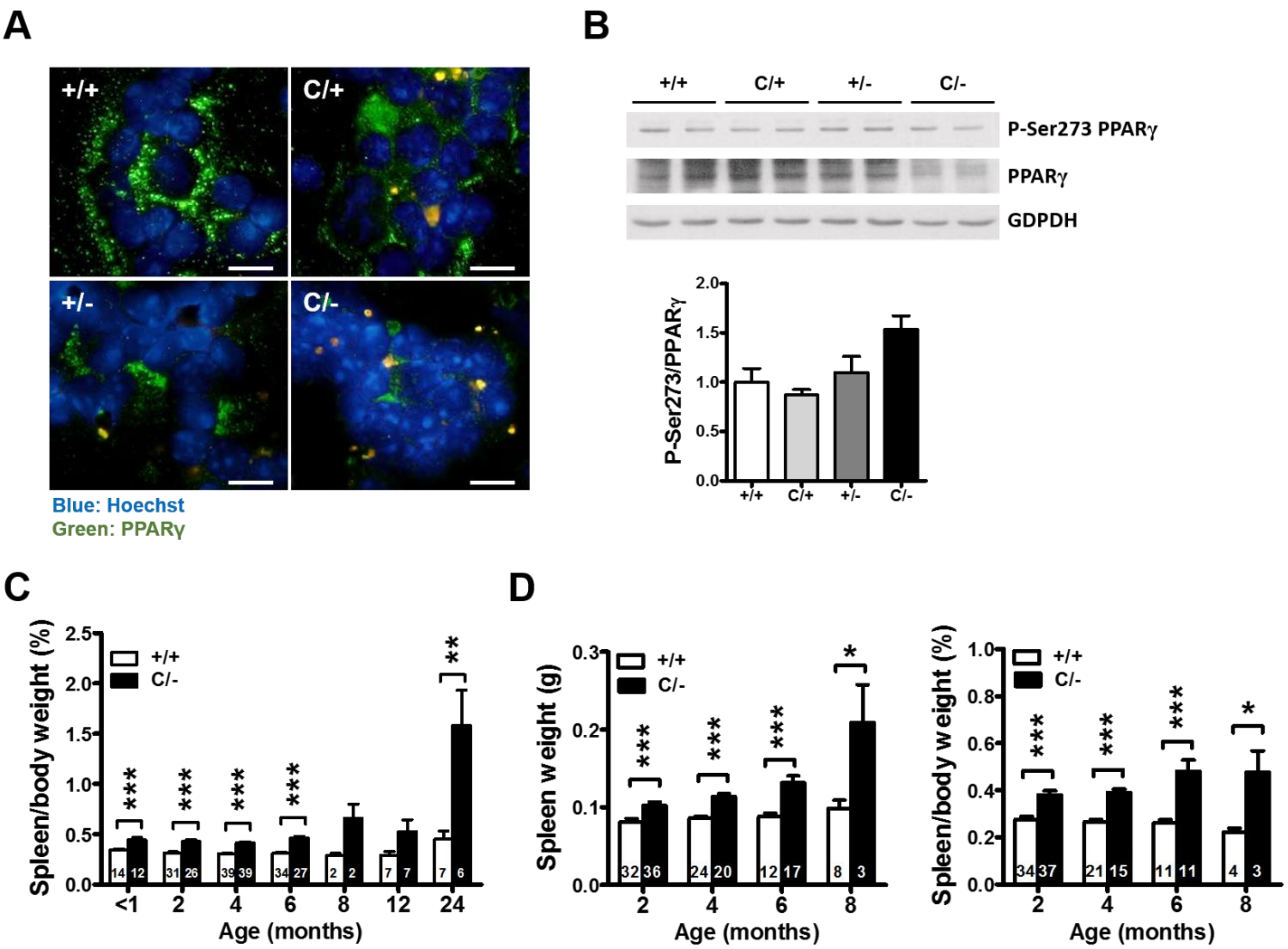

Supplemental Figure S1. Spleen enlargement in PPARγ hypomorphic mice. (A) Immunofluorescent staining of PPARγ (*green*) in splenocytes. The Hoechst nuclear counterstain appears *blue*. Scale bar, 20 μm (B) Ser273 phosphorylation of PPARγ in splenocytes was determined by immunoblotting using antibodies against P-Ser273 PPARγ and PPARγ. (C) Spleen-to-body weight ratio in female mice. (D) Spleen weight and spleen-to-body weight ratio in male mice. Numbers inside bars indicate the number for each group. **p* <0.05; ***p* <0.01; ****p* <0.001.

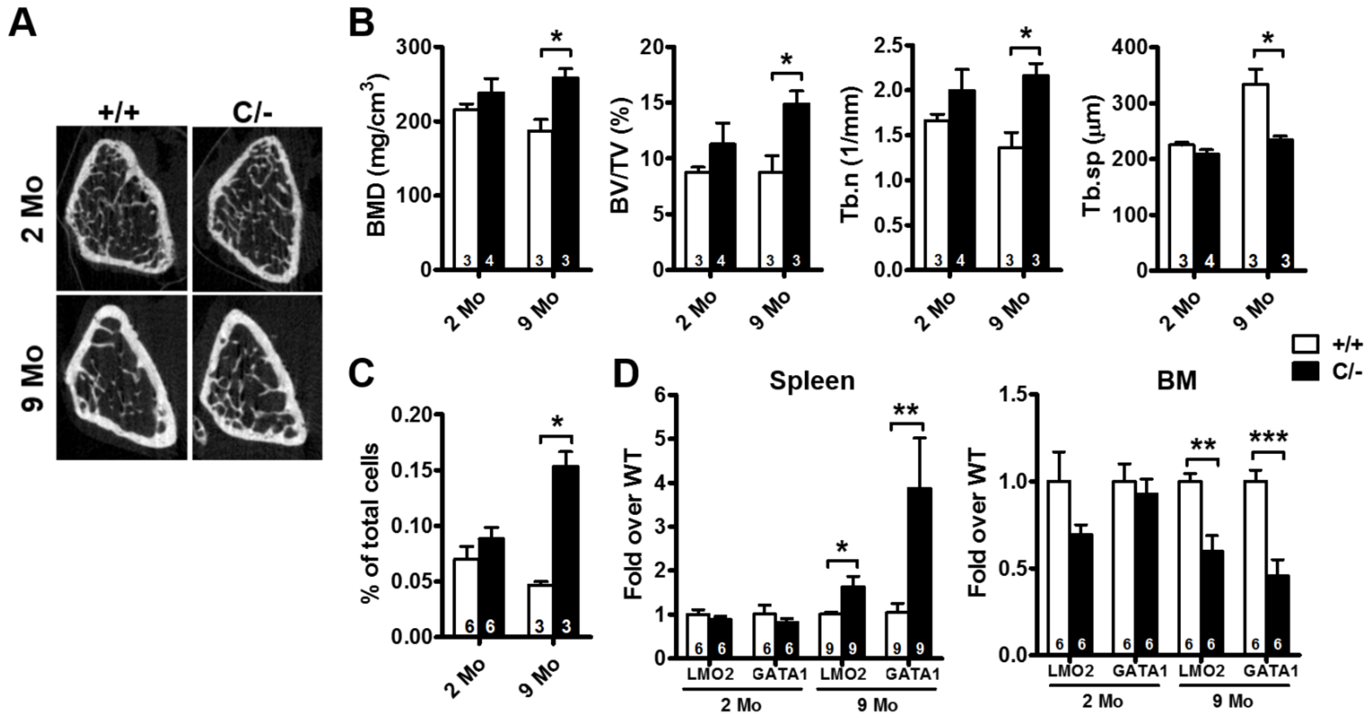

Supplemental Figure S2. Effects of PPARγ hypomorph on bone formation and hematopoietic stem cell relocation. (A) Micro-CT images of the distal femur metaphysis and (B) quantification of structural parameters of femoral metaphysis from 2-mo-old and 9-mo-old mice. Bone marrow density (BMD); trabecular volume (BV/TV); trabecular number (Tb.n); trabecular separation (Tb.sp). (C) Flow cytometric analysis of hematopoietic stem cells (HSCs) in the spleen of 2-mo-old and 9-mo-old mice. (D) Expression of transcription factors regulating early hematopoiesis in the spleen and bone marrow of 2-mo-old and 9-mo-old mice. LMO2, LIM domain only 2; GATA1, GATA-binding protein 1. Numbers inside bars indicate the number for each group. *p <0.05; **p <0.01; ***p <0.001.

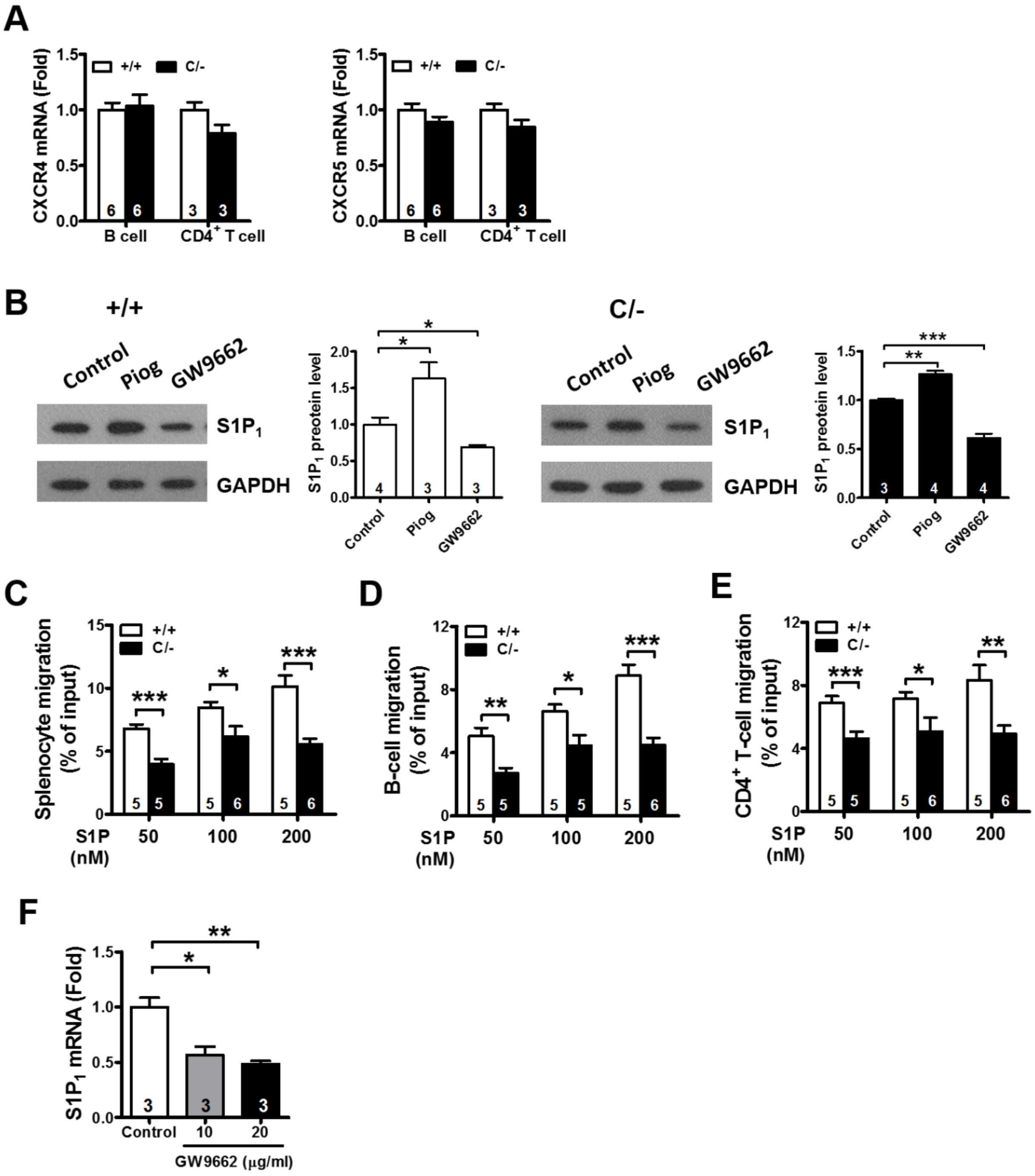

Supplemental Figure S3. Migration ability of splenocytes toward S1P and $S1P_1$ expression after modulation of PPARγ activity. (A) CXCR4 and CXCR5 mRNA levels in B cells and $CD4^+$ T cells from 3-mo-old mice. (B) $S1P_1$ protein levels in WT and *Pparg*$^{C/-}$ splenocytes after treatment with 80 μM Piog and 20 μM GW9662 for 24 hours. Migration of (C) splenocytes, (D) B cells and (E) $CD4^+$ T cells in response to different concentrations of S1P in Transwell migration assays. *p <0.05; **p <0.01; ***p <0.001. Two-way ANOVA for splenocyte migration in panel (C) shows effects of genotype (p <0.001) and S1P dosage (p <0.01). Two-way ANOVA for B-cell migration in panel (D) shows effects of genotype (p <0.001) and S1P dosage (p <0.001) with an interaction between these factors (p <0.05). Two-way ANOVA for $CD4^+$ T-cell migration in panel (E) shows effect of genotype (p <0.001). (F) $S1P_1$ expression in mouse embryonic fibroblasts treated with GW9662 (10 and 20 μg/ml) for 24 hours. Numbers inside bars indicate the number for each group.

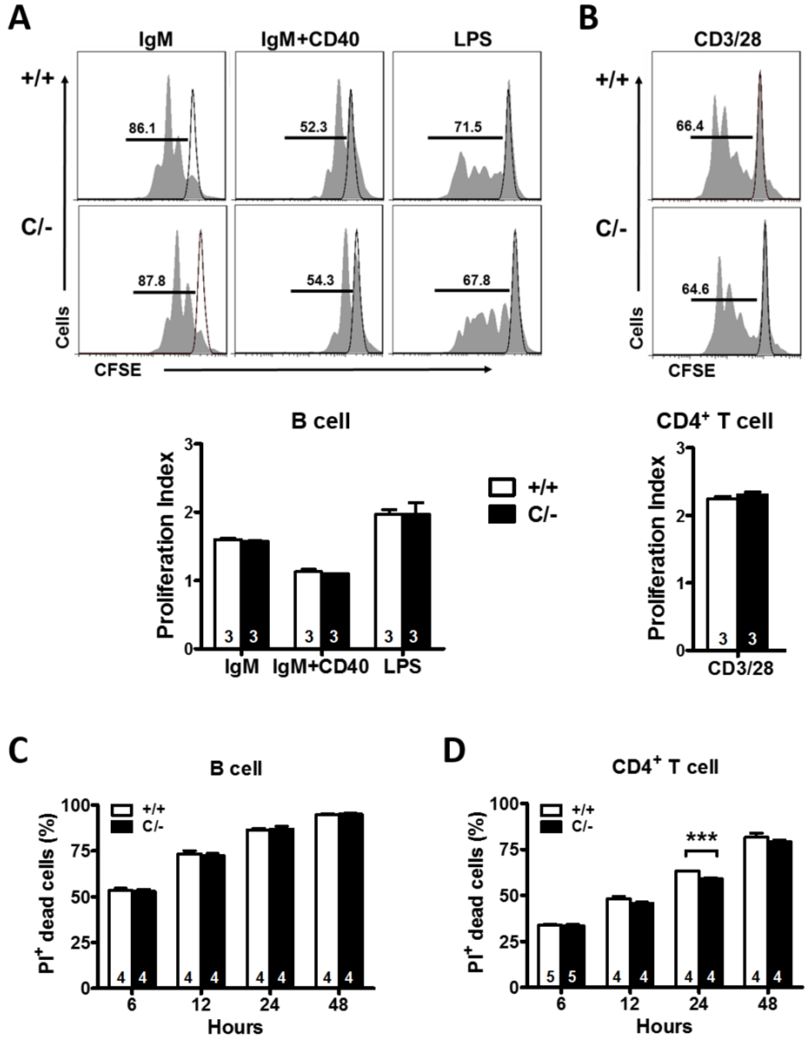

Supplemental Figure S4. In vitro cell proliferation and apoptosis. (A) CFSE-labeled B cells stimulated with anti-IgM (30 µg/ml) antibodies, a combination of anti-IgM (15 µg/ml) and anti-CD40 (10 µg/ml) antibodies, and LPS (10 µg/ml). (B) CFSE-labeled $CD4^+$ T cells stimulated with anti-CD3/CD28 (4 µg/ml) antibodies. Proliferation was analyzed by flow cytometry and the proliferation index was quantified by FlowJo software. Numbers inside bars indicate the number for each group. Percentages of propidium iodide (PI)-positive (C) B cells and (D) $CD4^+$ T cells cultured in the serum-free medium. B cells and $CD4^+$ T cells were isolated from 3-mo-old mice. Numbers inside bars indicate the number for each group.

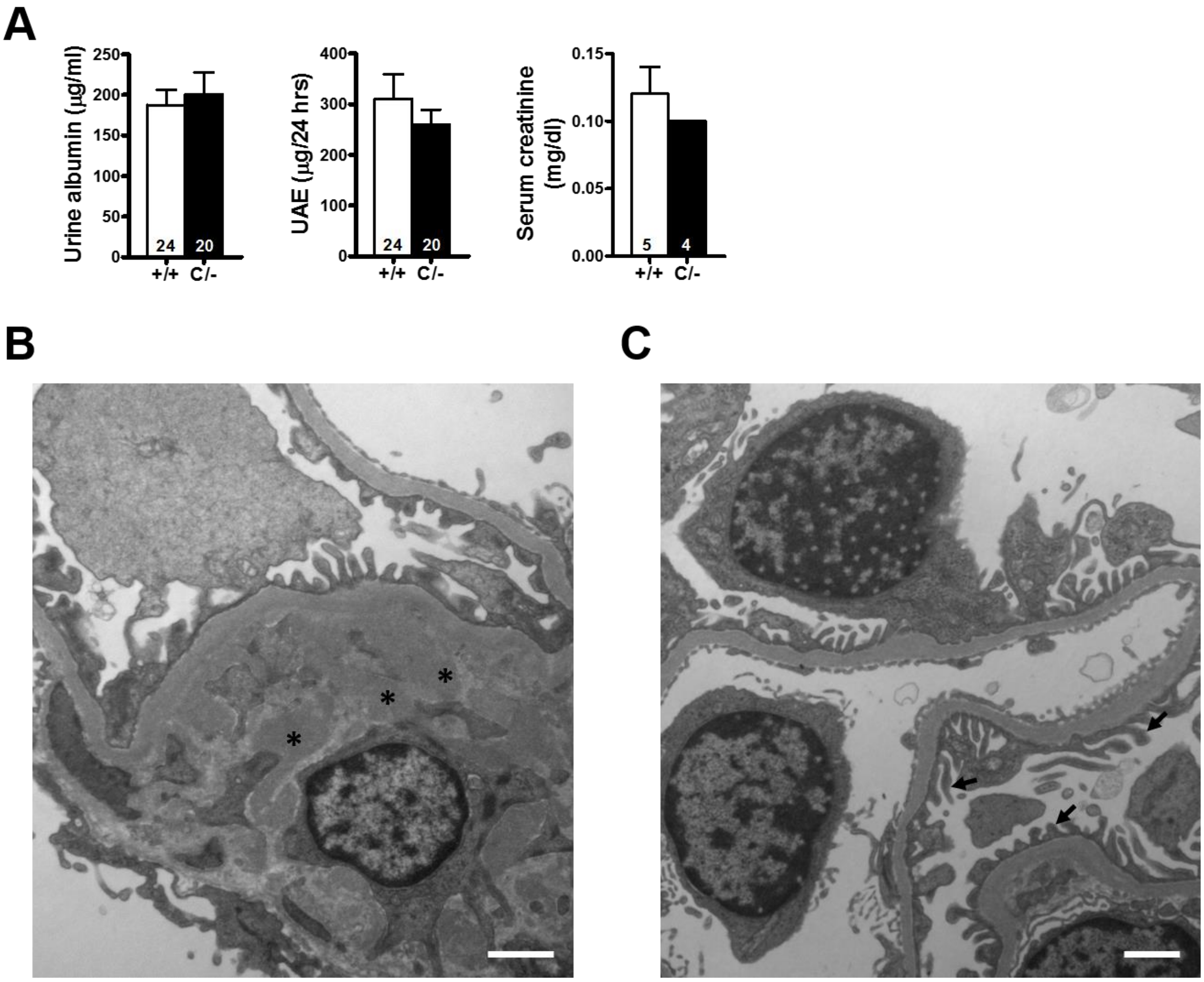

Supplemental Figure S5. Normal kidney function but electron-dense deposits in mesangial cells of *Pparg*$^{C/-}$ kidney. (A) Urine albumin concentration, daily urinary albumin excretion (UAE), and serum creatinine levels of 14-mo-old mice. Numbers inside bars indicate the number for each group. (B–C) Transmission electron microscopy of glomeruli. *Asterisks* show electron-dense deposits in the mesangium, and *black arrows* indicate normal podocyte foot processes. Scale bar, 1 µm.

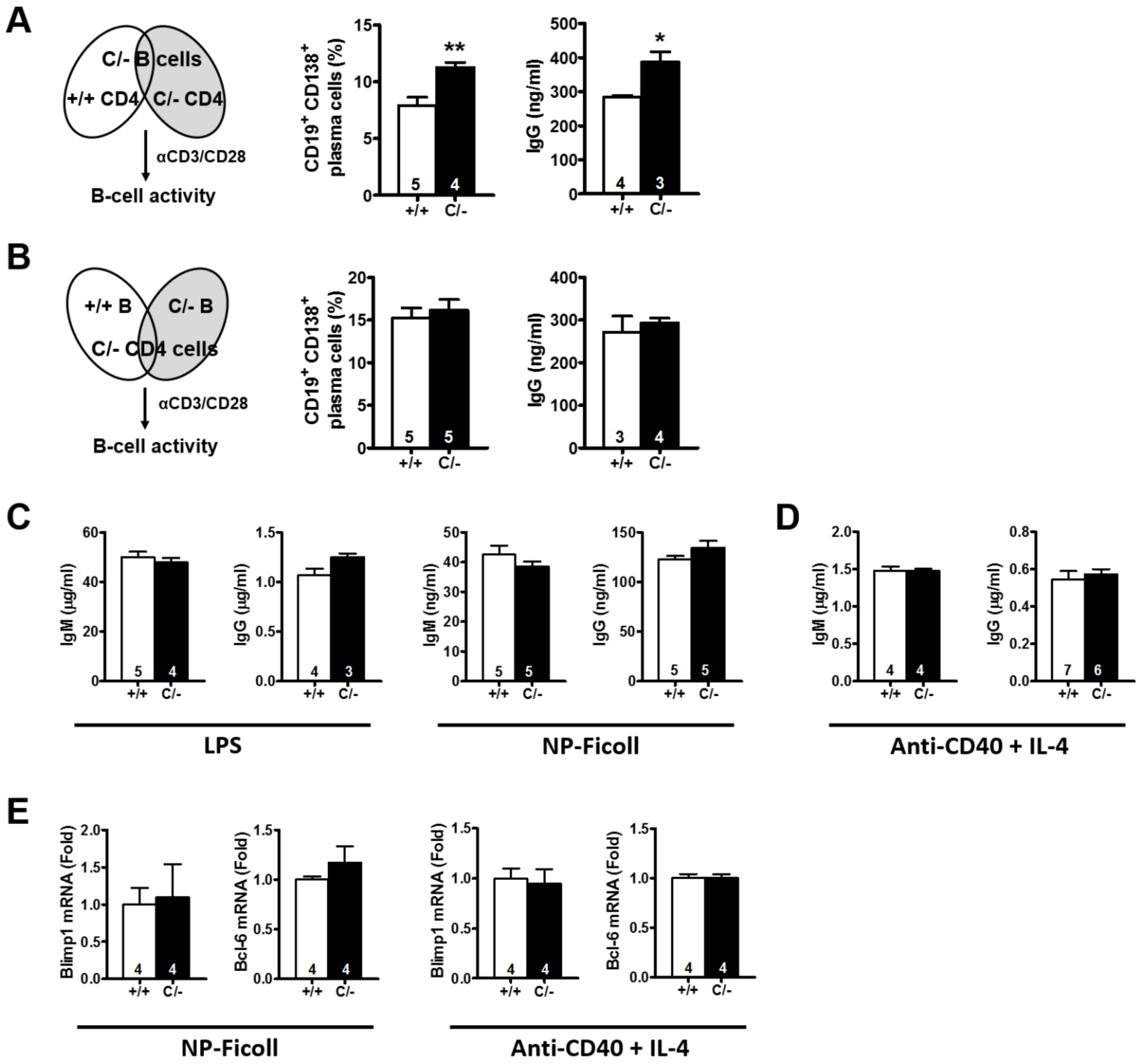

Supplemental Figure S6. In vitro B-cell response. (A) Coculture of *Pparg*$^{C/-}$ B cells with WT or *Pparg*$^{C/-}$ CD4^{+} T cells. Percentages of plasma cells (CD19^{+} CD138^{+}) gating from B cells (CD19^{+}) measured by flow cytometry after anti-CD3/CD28 stimulation for 3 d, and IgG production in the medium measured after anti-CD3/CD28 stimulation for 7 d. (B) Coculture of *Pparg*$^{C/-}$ CD4^{+} T cells with WT or *Pparg*$^{C/-}$ B cells, the same parameters as in A were measured. IgM and IgG production from WT or *Pparg*$^{C/-}$ B cells after stimulations with (C) LPS (5 μg/ml), NP-Ficoll (10 μg/ml), or (D) anti-CD40 (5 μg/ml) plus IL-4 (25 ng/ml) for 5 d. B cells were isolated from 4–6-mo-old mice. (E) Blimp1 and Bcl-6 mRNA levels of B cells in response to NP-Ficoll (10 μg/ml) or anti-CD40 (5 μg/ml) plus IL-4 (25 ng/ml) for 3 d. Numbers inside bars indicate the number for each group. *p <0.05 and **p <0.01.

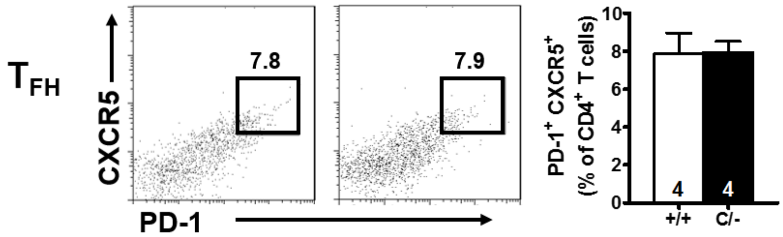

Supplemental Figure S7. Analysis of Tfh cells in the spleen of older WT and *Pparg*$^{C/-}$ mice. Flow cytometric analysis of the expression of PD-1 and CXCR5 on the splenic CD4$^+$ T cells. Numbers inside bars indicate the number for each group.

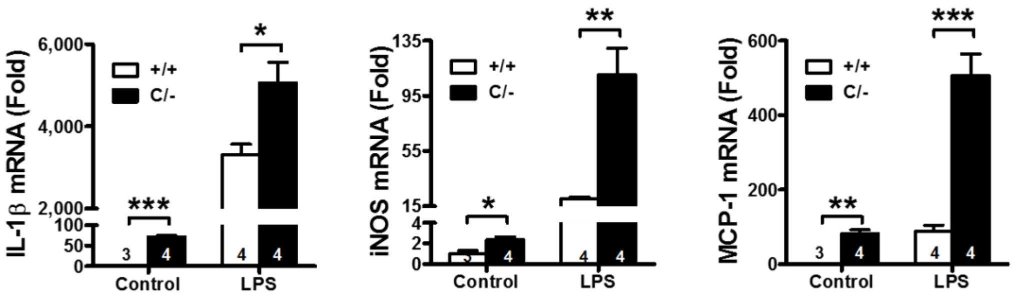

Supplemental Figure S8. Macrophage activation in WT and *Pparg*$^{C/-}$ mice. Basal and LPS-stimulated mRNA levels of IL-1β, iNOS and MCP-1 in peritoneal macrophages. LPS (500 ng/ml) was stimulated for 3.5 hours. Numbers inside bars indicate the number for each group. *p <0.05; **p <0.01; ***p <0.001.

Table SI Flow cytometric analyses of splenic cells from younger WT and *Pparg*$^{C/-}$ mice

	WT	*Pparg*$^{C/-}$	*P*-value
	**% of splenic cells**		
CD19^{+}	58.80 ± 1.89	58.38 ± 0.84	0.844
CD3^{+}	33.84 ± 1.73	31.75 ± 1.24	0.366
CD11C^{+}FSC^{+}	2.48 ± 0.53	1.93 ± 0.31	0.380
Gr-1^{-}CD11b^{+}F4/80^{+}	3.43 ± 0.53	2.72 ± 0.48	0.371
**B-cell subtypes**	**% of splenic CD19^{+} B cells**		
IgM^{high}IgD^{low}	19.53 ± 1.40	18.05 ± 1.61	0.514
IgM^{high}IgD^{high}	14.38 ± 0.34	13.51 ± 0.90	0.400
IgM^{low}IgD^{high}	44.48 ± 1.14	48.20 ± 1.77	0.126
CD21^{low}CD23^{+}	68.41 ± 1.17	69.90 ± 0.61	0.306
CD21^{high}CD23^{-}	13.00 ± 0.87	10.27 ± 0.78	0.058
**Plasma cells**	**% of splenic cells**		
CD19^{low}CD138^{+}	1.45 ± 0.20	1.57 ± 0.44	0.575
**Germinal center B cells**	**% of splenic cells**		
B220^{+}PNA^{hi}	1.07 ± 0.11	0.86 ± 0.21	0.424
**T cell subtypes**	**% of splenic CD3^{+} T cells**		
CD4^{+}	52.94 ± 4.94	59.61 ± 3.53	0.314
CD8^{+}	47.50 ± 0.87	49.94 ± 3.04	0.414
**CD62L and CD44 profile**	**% of splenic CD4^{+} T cells**		
CD62L^{hi}CD44^{low}	69.43 ± 2.36	73.95 ± 2.78	0.261
CD62L^{hi}CD44^{hi}	16.13 ± 1.79	14.43 ± 1.78	0.526
CD62L^{low}CD44^{hi}	7.19 ± 0.17	6.73 ± 0.62	0.496

Four 5-mo-old mice per group were analyzed and the data are presented as the average ± SEM for each group.

Table SII Flow cytometric analyses of splenic cells from older WT and *Pparg*$^{C/-}$ mice

	WT	*Pparg*$^{C/-}$	*P*-value
	% of splenic cells		
CD19^{+}	45.08 ± 1.78	50.08 ± 0.71	0.197
CD3^{+}	41.48 ± 2.56	35.38 ± 2.36	0.124
CD11C^{+}FSC^{+}	3.28 ± 0.50	3.63 ± 0.37	0.591
Gr-1^{-}CD11b^{+}F4/80^{+}	6.86 ± 1.65	6.07 ± 1.13	0.704
**B-cell subtypes**	**% of splenic CD19^{+} B cells**		
IgM^{high}IgD^{low}	13.44 ± 0.32	13.72 ± 0.88	0.772
IgM^{high}IgD^{high}	11.74 ± 1.46	13.70 ± 1.34	0.320
IgM^{low}IgD^{high}	42.98 ± 2.51	38.92 ± 3.23	0.350
CD21^{low}CD23^{+}	62.22 ± 2.19	65.04 ± 0.74	0.257
CD21^{high}CD23^{-}	13.42 ± 0.97	11.64 ± 0.49	0.140
**Plasma cells**	**% of splenic cells**		
CD19^{low}CD138^{+}	0.65 ± 0.75	0.61 ± 0.15	0.860
**Germinal center B cells**	**% of splenic cells**		
B220^{+}PNA^{hi}	2.23 ± 0.38	2.09 ± 0.21	0.760
**T-cell subtypes**	**% of splenic CD3^{+} T cells**		
CD4^{+}	32.51 ± 2.14	35.56 ± 2.98	0.429
CD8^{+}	30.29 ± 2.17	22.52 ± 3.44	0.073
**CD62L and CD44 profile**	**% of splenic CD4^{+} T cells**		
CD62L^{hi}CD44^{low}	55.72 ± 4.54	48.30 ± 5.07	0.307
CD62L^{hi}CD44^{hi}	20.30 ± 2.16	26.28 ± 3.10	0.152
CD62L^{low}CD44^{hi}	8.76 ± 0.86	9.51 ± 0.35	0.437

Five 14-mo-old mice per group were analyzed and the data are presented as the average ± SEM for each group.

Table SIII Complete blood cell counts in WT and *Pparg*$^{C/-}$ mice

Genotype	WBC (K/μl)	Lymphocyte (K/μl)	CD19^{+} B cell (K/μl)	CD3^{+} T cell (K/μl)
**WT**	9.56 ± 0.67	7.29 ± 0.61	4.32 ± 0.33	2.23 ± 0.34
***Pparg*$^{C/-}$**	9.22 ± 0.46	7.21 ± 0.30	4.46 ± 0.33	1.90 ± 0.34

Four 4-mo-old mice per group were analyzed and the data are presented as the average ± SEM for each group.
